# Cerebral gray matter volume changes in patients with anti-N-methyl-D-aspartate receptor encephalitis: A voxel-based morphometry study

**DOI:** 10.3389/fneur.2022.892242

**Published:** 2022-07-25

**Authors:** Qijia Long, Zongxia Lv, Jingyuan Zhao, Ke Shi, Chunyan Li, Binglin Fan, Jinou Zheng

**Affiliations:** Department of Neurology, The First Affiliated Hospital of Guangxi Medical University, Nanning, China

**Keywords:** anti-N-Methyl-D-aspartate receptor encephalitis, voxel-based morphometry, gray matter volume, cognitive function, MRI

## Abstract

**Background:**

Anti-N-methyl-D-aspartate receptor (NMDAR) encephalitis is an autoimmune disease with typical clinical features. Whether and how cerebral gray matter structural damage inherent to the disorder affects cognitive function in patients is still unclear. Therefore, this study aimed to explore the changes in cerebral gray matter volume and whether these alterations contribute to cognitive impairment and mood disorders.

**Methods:**

Forty patients with anti-NMDAR encephalitis and forty healthy controls (HCs) matched for gender, age, and education were recruited. All participants underwent attention network tests (ANT), neuropsychological tests and magnetic resonance imaging (MRI). Voxel-based morphological analysis (VBM) and correlation analysis was performed on all participants. Finally, according to the course of disease, patients were divided into two groups: NMDARE_SD (short duration; course ≤ 2 years since diagnosis) and NMDARE_LD (long duration; course >2 years since diagnosis), to evaluate gray matter volume changes that differ as a function of disease course.

**Results:**

Compared to HCs, patients with anti-NMDAR encephalitis showed decreased executive control ability and lower MoCA score, while increased anxiety and depression as reflected by HAMA and HAMD24 scores (all *P* < 0.05). In VBM analysis, patients showed decreased gray matter volume in bilateral thalamus, left medial prefrontal cortex (mPFC_L), left superior temporal gyrus (STG_L), and left rectus gyrus. In the analysis stratified by disease course, the NMDARE_LD group exhibited decreased gray matter volume in the left precuneus and right posterior cerebellar lobe compared to the NMDARE_SD group.

**Conclusions:**

Patients with anti-NMDAR encephalitis have cognitive, executive, and emotional dysfunction, and the sites of gray matter atrophy are concentrated in the thalamus, frontal lobe, and temporal lobe. These abnormalities may be involved in the process of cognitive and affective dysfunction.Patients with different courses of anti-NMDAR encephalitis have different brain atrophy sites. These results may help to clarify the contradiction between clinical and imaging manifestations of anti NMDAR encephalitis, which is worthy of further longitudinal studies.

## Introduction

Anti-N-methyl-D-aspartate receptor (NMDAR) encephalitis is the most common type of autoimmune encephalitis (1), accounting for about 80% of autoimmune encephalitis cases (2). A recent study showed that the incidence of anti-NMDAR encephalitis is higher than that of any common viral encephalitis in patients under the age of 30 (3). The pathogenic mechanism may be the rapid and reversible decrease of NMDARs, which affects synaptic plasticity in neurons, resulting in the loss of NMDAR-mediated synaptic function and corresponding neurological dysfunction (4). The clinical manifestations of anti-NMDAR encephalitis include mental and behavioral abnormalities, memory and cognitive impairment, seizures, central hypoventilation, motor dysfunction, and even death in severe cases (5). The largest anti-NMDAR encephalitis cohort study to date showed that after 24 months of follow-up, 81% of patients had a good prognosis, 13% of patients relapsed, and 6% of patients died (6). In addition, even after recovery, patients often experience permanent neurological dysfunction, especially in cognitive areas such as memory, attention, and executive control function (7, 8).

The diagnosis of anti-NMDAR encephalitis mainly relies on typical clinical manifestations and detection of specific antibodies in cerebrospinal fluid or serum (9). Conventional magnetic resonance imaging (MRI) is often used to assist in diagnosis, and 33–55% of patients show abnormal brain MRI results (10). Evidence of brain damage is often observed in the medial temporal, frontal, and parietal cortex, but occasionally shows in the cerebellum, thalamus, basal ganglia, brainstem, and spinal cord (11). Numerous studies have reported structural and functional changes in multiple brain regions in patients with anti-NMDAR encephalitis based on functional MRI and diffusion tensor imaging, which are associated with cognitive impairment and other clinical symptoms (12). Common abnormalities reported in positron emission tomography (PET) studies include hypermetabolism in frontal, insular, and temporal areas, and hypometabolism in the parietal and occipital lobes (10, 13), indicating functional brain abnormalities in patients with anti NMDAR encephalitis. In the study of brain structure, some studies reported that patients with anti-NMDAR encephalitis had reversible diffuse brain atrophy (14), but one case report of a 25-year-old female patient showed that, despite a normal baseline MRI scan, the patient showed atrophy of the frontal cortex after 9 months of follow-up (15). Whether and how the cerebral gray matter structural damage affects cognitive function in patients is still unclear. Studying the effects of antibody-mediated NMDAR dysfunction on brain gray matter structure and its relationship with clinical symptoms provides a new perspective for understanding its underlying mechanisms.

With the continuous advancements of neuroimaging technology, more and more methods are being applied to the study of anti-NMDAR encephalitis. Among them, voxel-based morphological analysis (VBM) is a method of assessing brain structure at the voxel level. The analytical technology can quantitate changes in the gray and white matter signals and volume of local brain regions, thereby accurately characterizing the morphological changes in brain tissues. Compared with traditional region of interest (ROI) measurement, VBM can detect subtle brain structural abnormalities, thereby improving the statistical validity of morphological analysis (16). This method has been widely used in research on epilepsy, Parkinson's disease, Alzheimer's disease, and schizophrenia (17–20), which can provide strong evidence to explain the underlying pathophysiological mechanisms of disease. Our research results can complement the previous imaging studies on anti-NMDAR encephalitis.

Most of the current studies evaluating brain structural changes in patients with anti-NMDAR encephalitis are case reports, and there is a lack of studies implementing control groups. A recent study on the gray matter changes in autoimmune encephalitis, including anti-NMDAR encephalitis, showed that there were different atrophy patterns between patients with a disease duration of ≤ 2 years and those with a disease duration of >2 years (21). However, gray matter changes were not specifically assessed in patients with anti-NMDAR encephalitis with different courses of disease. Therefore, this study systematically evaluated cognitive, executive control, and emotional functions in conjunction with gray matter volume changes of anti-NMDAR encephalitis patients. The purpose of this study is to explore whether the damage to gray matter structure in patients with anti-NMDAR encephalitis is related to cognitive, executive, and emotional dysfunction, and whether there are differences in the pattern of gray matter atrophy in patients with different disease courses.

## Materials and methods

### Participants

In this study, 40 patients with anti-NMDAR encephalitis were recruited from the Department of Neurology of the first affiliated Hospital of Guangxi Medical University from June 2018 to June 2021, and 40 healthy controls (HCs) without neuropsychiatric disease matched for sex, age, and education were recruited from the community. According to the diagnostic criteria of anti-NMDAR encephalitis (22), the inclusion criteria for patients in this study included: (1) presenting with one or more of the following symptoms: abnormal mental behavior or cognitive impairment, seizures, autonomic nervous dysfunction or central hypoventilation, dyskinesia or involuntary movement, disturbance of consciousness, or speech disorder; (2) The cerebrospinal fluids of all patients were sent to an independent third-party commercial testing center for anti-NMDAR antibody detection using a cytometric bead array (CBA) commercial kit. All patients were right-handed and between 18 and 60 years old. Exclusion criteria included the presence of other neuropsychiatric disorders that may affect cognition, drug abuse, or contraindications for MRI.

To study the brain atrophy of patients with anti-NMDAR encephalitis with different disease courses, the patients were divided into two groups: those with short duration of disease (≤ 2 years since diagnosis: NMDARE_SD) and those with a long duration of disease (>2 years since diagnosis: NMDARE_LD). The duration of disease was calculated from the time when the patients were diagnosed with anti-NMDAR encephalitis until the day of the MRI scan.

The study protocol was approved by the First Affiliated Hospital of Guangxi Medical University Ethics Committee.

### Behavioral tasks and neuropsychological tests

Behavioral tasks and neuropsychological tests were performed on all subjects on the day of MRI scans, including the Attention Network Test (ANT) to assess executive function, the Montreal Cognitive Assessment test (MoCA) to assess general cognitive function, and the Hamilton anxiety scale (HAMA) and Hamilton Depression scale-24 (HAMD_24_) to assess anxiety and depression, respectively. The clinical scales were validated for the native language of the patients.

Executive control (EC) function for all subjects was evaluated by ANT software (23). Testing was conducted in a quiet environment to ensure minimal disturbance to the subjects. Before starting the test, participants performed 24 training trials under guidance to familiarize themselves with the procedure. The formal examination was divided into three modules, with each module containing 32 times, for a total of 96 trials. The whole exam lasted approximately 25 min. At the beginning of the experiment, a fixation cross (+) was presented in the middle of the computer screen, followed by presentation of the target stimulus (five arrows arranged horizontally) above or below the cross for 1,700 ms. The target stimulus contained three types of arrows: non-conflict (neutral, no interference, only the middle arrow points left and right, and four unpainted “-” on the side), consistent (five arrows point to the same, all to the left or right) and inconsistent (the middle arrow points a direction inconsistent with the direction of the four surrounding arrows). Participants were positioned 60 cm from the front of the screen with their fingers placed on the left and right mouse buttons. They were instructed to judge the direction of the middle arrow and press the corresponding mouse button (i.e., the left mouse button for arrows pointing left, the right mouse button for arrows pointing right). The system automatically identified and recorded reaction time (RT) for each trial. After a choice was made or the time limit expired, the next trial began.

The executive control score was measured as follows:


$$\begin{array}{l} \text{EC}={\text{RT}}_{\text{inconsistent}}-{\text{RT}}_{\text{consistent}} \end{array}$$

A higher EC scores indicates worse executive function.

### MRI data acquisition

MRI data were acquired using an Achieva 3.0-T MRI scanner with a 12-channel head coil (Philips, Amsterdam, Netherlands). During the scanning process, rubber earplugs were used to reduce noise and foam pads used to fix the head to minimize potential motion artifacts. Subjects were instructed to stay awake and relaxed, think of nothing, and keep their eyes closed. Fast gradient echo sequences were used to obtain T1W structural images. The scanning parameters were as follows: repetition time (TR) = 7.8 ms, echo time (TE) = 3.4 ms, slice thickness = 1.4 mm, layer spacing = 1.4 mm, flip angle = 20°, layer number = 176.0 mm, visual field = 240 × 240 mm, voxel size = 1 × 1 × 1 mm, matrix = 256 × 256, and scanning time = 5 min 49 s. Moreover, no obvious lesions were identified in all T1 images after visually checked by two experience radiologists.

### MRI data preprocessing

SPM8 software based on Matlab2013b was used to preprocess T1-weighted (T1W) images for all subjects. The steps included conversion of all images from DICOM format to NifTI format, normalization to standard montreal neurological institute (MNI) space, and smoothing with an 8-mm FWHM kernel. At the whole brain level, a voxel-wise two-sample *T*-test was performed to compare gray matter volume differences between patient and control groups, with age, gender, education, and whole brain volume as covariates, separately [*P* < 0.05, family-wise error (FWE) corrected].

To further analyze whether patterns of gray matter atrophy differ as a function of disease course, patients were dichotomized into NMDARE_SD and NMDARE_LD groups. A voxel-wise two-sample *T*-test was performed to identify brain regions with different gray matter volume between the two groups, with age, gender, education, and whole brain volume as covariates, separately (*P* < 0.001 uncorrected).

### Statistical analysis

General demographic data were analyzed by SPSS25.0 software. The data are reported in the form of mean ± standard deviation. Categorical variables (e.g., gender) were compared by Chi^2^ test. Group differences in demographic and clinical characteristics such as age, education level, MoCA, HAMA, HAMD_24_ and EC scores were assessed by two-sample *T*-test. Gray matter volumes of patients with anti-NMDAR encephalitis in those brain areas which showed significant differences in the patient group vs. HCs group, and NMDARE_SD vs. NMDARE_LD were extracted, and Pearson correlation analysis was performed to determine the relationship between gray matter volume and disease duration, MoCA, HAMA, HAMD_24_ and EC scores. *P* < 0.05 indicated a statistically significant difference.

## Results

### Comparison between patients and HCs

#### Demographic data and scale scores

Forty patients with anti-NMDAR encephalitis were enrolled in this study, including 16 males and 24 females. Among them, 22 patients had a disease duration of ≤ 2 years, while 18 patients had a disease duration of >2 years. The patients showed typical clinical symptoms, including headache, fever, psychiatric symptoms, dyskinesias/movement disorders, and seizures. Six patients (15%) had experienced a relapse and were hospitalized again. All patients received standard immunotherapy after the onset of the disease and had completed the course of immunotherapy prior to study enrollment. The detailed clinical data are shown in Table 1. When evaluating the clinical scale, the patient with cognitive and emotional disorders should be admitted to the Department of Neurology and Psychology in time.

**Table 1:** Clinical data of patients with anti-NMDAR encephalitis.

**Characteristic/symptoms**	***n***	**%**
Total	40	100
Sex		
Male	16	40
Female	24	60
Disease duration ≤ 2 years	22	55
Disease duration > 2 years	18	45
Symptom presentation		
Headache	8	20
Fever	9	22.5
Psychiatric symptoms	31	77.5
Dyskinesias and movement disorders	13	32.5
Disorders of consciousness	15	37.5
Seizures	32	80
First line therapy (Corticosteroid, intravenous immunoglobulin, plasma exchange)	40	100
Second-line therapy (Cyclophosphamide)	2	5
Relapses	6	15

There were no significant group differences in age, sex, or years of education, but the MoCA scores in the patients were significantly lower than those in the HCs. The HAMD24 and HAMA scores, and executive control scores were significantly higher in patients compared to HCs, indicating poorer function. The demographic details and neuropsychological test data are shown in Table 2.

**Table 2:** Comparison of demographic details and neuropsychological tests data between the Patients and healthy controls.

**Characteristics**	**Patients** **(*n* = 40)**	**Healthy controls** **(*n* = 40)**	***P*** **value**
Gender (M/F)	16/24	14/26	0.64*
Age (years)	27.33 ± 9.31	28.65 ± 7.78	0.49
Education (years)	13.38 ± 3.65	14.60 ± 3.02	0.11
Age of onset (years)	25.78 ± 9.40	–	–
Disease duration (months)	19.55 ± 14.42	–	–
MoCA scores	25.10 ± 4.95	28.58 ± 1.74	<0.001
HAMD_24_ scores	5.00 ± 5.17	1.35 ± 1.79	<0.001
HAMA scores	3.30 ± 3.10	0.90 ± 1.63	<0.001
Executive control scores (ms)	116.74 ± 63.77	89.00 ± 29.05	0.02

P <0.05 was considered statistically significant. ^*^χ^2^ test for sex (n). Independent t-test for the other data (means ± SD). M, male; F, female; MoCA, montreal cognitive assessment test; HAMD24, hamilton depression scale-24; HAMA, hamilton anxiety scale.

#### VBM analysis

**Figure 1:**
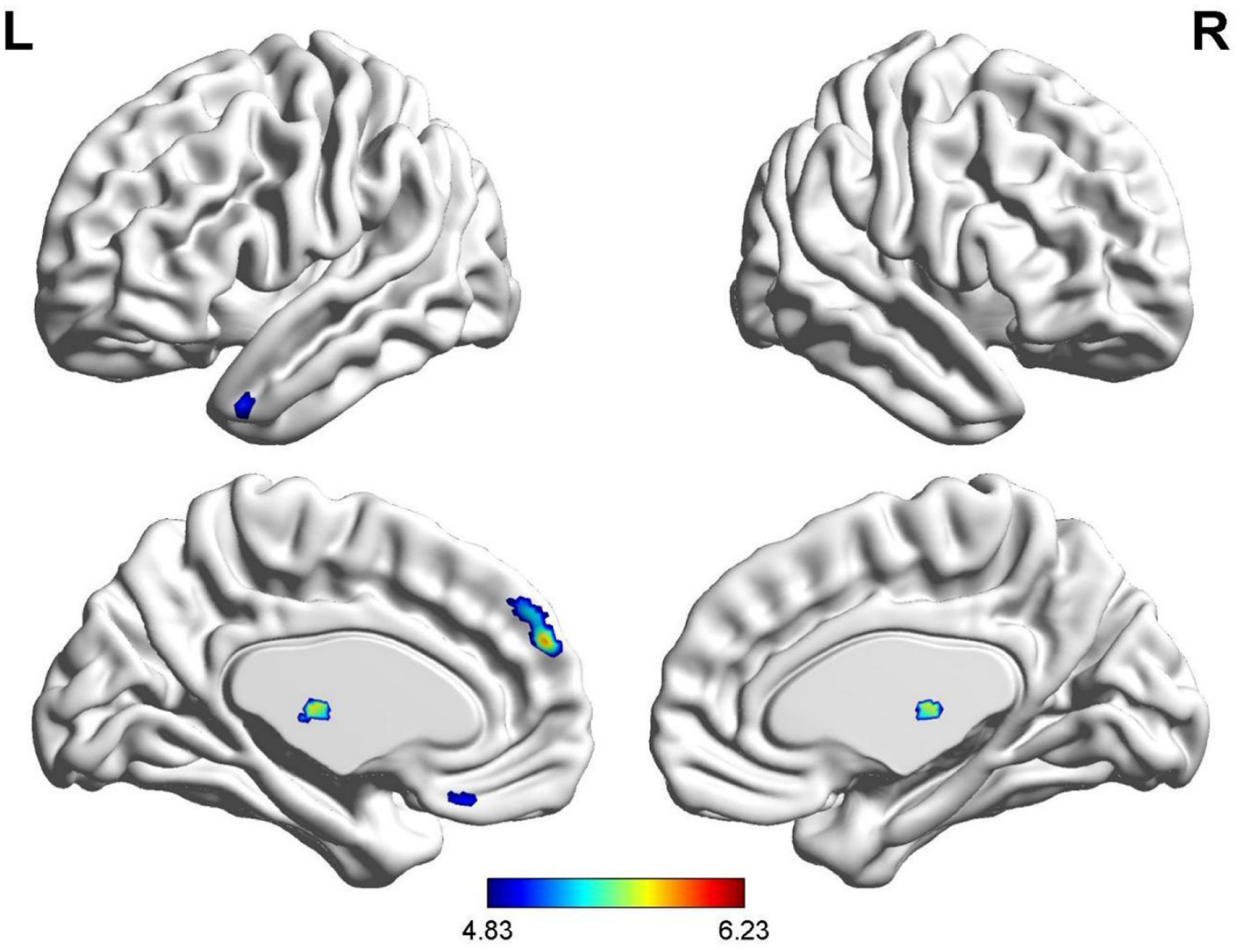
Brain regions with a significantly decreased volume of gray matter in anti-NMDAR encephalitis patients compared to healthy controls.

Compared with HCs, gray matter volume in bilateral thalamus, left medial prefrontal cortex (mPFC_L), left superior temporal gyrus (STG_L), and left rectus gyrus was lower in patients with anti-NMDAR encephalitis. No elevations in gray matter volume in patients relative to controls were observed. See Table 3, Figure 1 for details.

**Table 3:** Brain regions with a significantly decreased volume of gray matter in patients compared to healthy controls.

**Brain regions**	**Cluster**	**Peak MNI**			***t*** **-value**
		***X***	***Y***	***Z***	
Thalamus_L	370	6	−9	2	6.15
Thalamus_R	549	−7	−9	2	6.01
Medial prefrontal cortex_L	355	−1	53	27	6.23
Superior temporal gyrus_L	75	−48	−6	0	5.65
Rectus gyrus_L	102	−4	27	−21	5.18

*MNI, montreal neurological institute; L, left; R, right. P <0.05 family-wise error (FWE) correction*.

### Anti-NMDAR encephalitis subgroup analysis

#### Demographic data and scale scores

There were no significant differences in age, sex, or years of education between the NMDARE_SD and NMDARE_LD groups, and there were no significant differences in cognitive function (MoCA), EC scores, or mental and emotional scores (HAMD_24_, HAMA). The detailed clinical data are shown in Table 4.

**Table 4:** Comparison of clinical data between the NMDARE_SD and NMDARE_LD.

**Characteristics**	**NMDARE_LD (*n* = 18)**	**NMDARE_SD (*n* = 22)**	***P*** **value**
Gender (M/F)	10/8	6/16	0.07*
Age (years)	28.11 ± 10.36	26.68 ± 8.55	0.64
Education (years)	13.17 ± 4.05	13.55 ± 3.38	0.75
Age of onset (years)	25.50 ± 10.55	26.00 ± 8.47	0.87
Disease duration (months)	32.89 ± 10.41	8.64 ± 4.61	–
MoCA scores	25.28 ± 4.54	24.50 ± 5.34	0.63
HAMD_24_ scores	4.28 ± 3.75	5.59 ± 6.12	0.43
HAMA	2.56 ± 2.46	3.91 ± 3.48	0.17
Executive control scores(ms)	124.35 ± 71.91	122.82 ± 56.99	0.94

NMDARE_SD, Anti-NMDAR encephalitis with short disease duration (≤ 2 years since diagnosis); NMDARE_LD, Anti-NMDAR encephalitis with long disease duration (>2 years since diagnosis); M, male; F, female; MoCA, montreal cognitive assessment test; HAMD_24_, hamilton depression scale-24; HAMA, hamilton anxiety scale. p <0.05 was considered statistically significant. ^*^χ^2^ test for sex (n). Independent t-test for the other data (means ± SD).

#### VBM analysis

**Figure 2:**
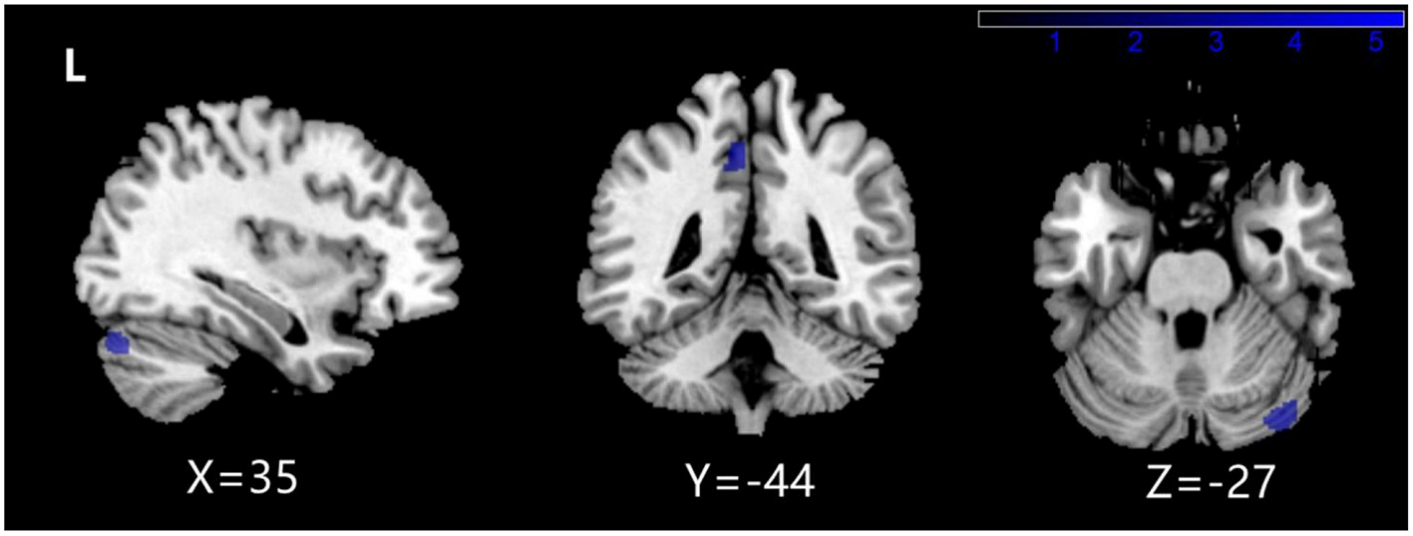
Brain regions with a significantly decreased volume of gray matter in NMDARE_LD compared to NMDARE_SD.

The two-sample *T* test results between NMDARE_SD and NMDARE_LD groups showed that NMDARE_LD patients had significantly more atrophy in the left precuneus and right posterior cerebellum (PCL_R) (*P* < 0.001 uncorrected; Figure 2, Table 5). No brain regions had showed increased volume in the NMDARE_LD group compared to the NMDARE_SD group.

**Table 5:** Brain regions with a significantly decreased volume of gray matter in NMDARE_SD compared to NMDARE_LD.

**Brain regions**	**Cluster**	**Peak MNI**			***t*** **-value**
		***X***	***Y***	***Z***	
Posterior Cerebellar Lobe_R	411	39	−79	−28	4.10
Precuneus _L	176	−4	−43	48	4.30

*NMDARE_SD, Anti-NMDAR encephalitis with short disease duration (≤ 2 years since diagnosis); NMDARE_LD, Anti-NMDAR encephalitis with long disease duration (>2 years since diagnosis); MNI, montreal neurological institute; L, left; R, right. P <0.001 uncorrected*.

### Correlation analysis

**Figure 3:**
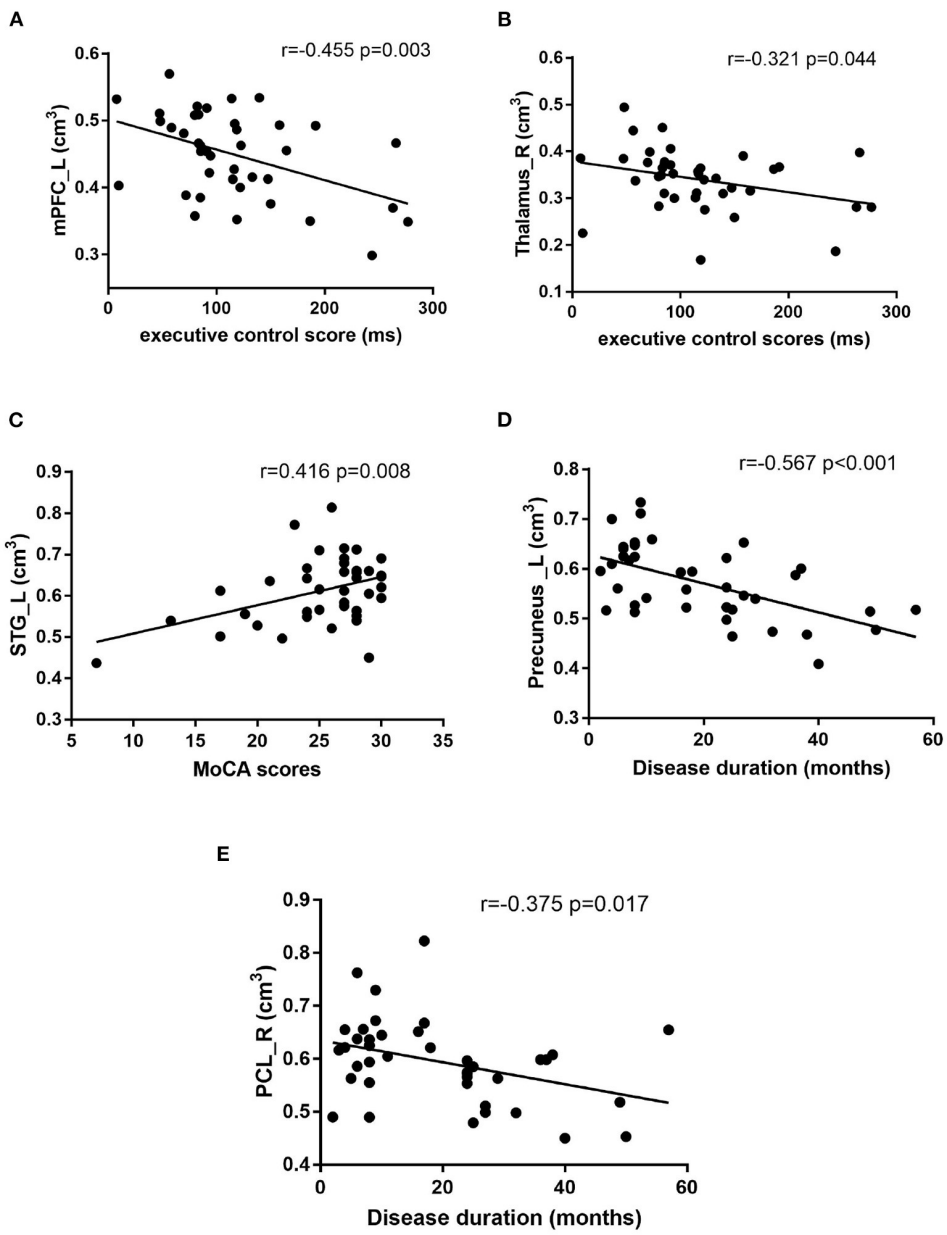
Correlation analysis: **(A)** The executive control (EC) scores was negatively correlated with the left medial prefrontal cortex (mPFC_L) volume (*r* = −0.455 *p* = 0.003), **(B)** The EC scores was negatively correlated with right thalamus volume (*r* = −0.321 *p* = 0.044), **(C)** The Montreal Cognitive Assessment test (MoCA) scores was positively correlated with left superior temporal gyrus (STG_L) volume (*r* = 0.416 *p* = 0.008), **(D)** The disease duration was negatively correlated with the left precuneus volume (*r* = −0.567 *p* < 0.001), **(E)** The disease duration was negatively correlated with the right posterior cerebellum (PCL_R) volume (*r* = −0.375 *p* = 0.017).

Pearson correlation analysis was performed to assess the relationships between the gray matter volume of bilateral thalamus, mPFC_L, STG_L, left straight gyrus, left precuneus, PCL_R, and disease duration, MoCA, HAMA, HAMD_24_, EC score. As shown in Figure 3, EC score was negatively correlated with gray matter volume in mPFC_L (*r* = −0.455, *P* = 0.003) and the right thalamus (*r* = −0.321, *P* = 0.044). STG_L gray matter volume was positively correlated with MoCA score (*r* = 0.416, *P* = 0.008), the course of the disease was both negatively correlated with left precuneus (*r* = −0.567, *P* < 0.001), and PCL_R (*r* = −0.375, *P* = 0.017). There were no significant correlations between the gray matter volume of these brain regions and HAMA and HAMD_24_ scores (P>0.05).

## Discussion

In this study, VBM was used to investigate gray matter morphological changes in 40 patients with anti-NMDAR encephalitis and 40 HCs. The results showed that patients exhibited decreased gray matter volume in several brain regions compared with HCs, including the bilateral thalamus, mPFC_L, STG_L, and left rectus gyrus. When stratifying patients into NMDARE_SD and NMDARE_LD groups, we found that, compared with NMDARE_SD patients, NMDARE_LD patients had more obvious atrophy in the left precuneus and PCL_R. compared with NMDARE_SD patients. These results indicate that patients with anti-NMDAR encephalitis have partial gray matter atrophy, and patients with different courses have different atrophy patterns. Further correlation analyses showed that these changes were related to patients' cognitive dysfunction, including executive control.

The thalamus is the relay center of nerve signal transduction processes, which is involved in sensory and motor functions. Thalamic neurons receive sensory or stimulus signals from the rest of the body and project to the cerebral cortex through nerve fibers (thalamocortical tract). The formation of the cortico-striato-thalamo-cortical loop is an important neural structure in the cerebral cortex for identification of sensory properties, localization, and emotional responses to sensory stimuli. It plays an important role in maintaining alertness, emotion, and other cognitive functions (24). In this study, we found that the bilateral thalamic gray matter volume in patients with anti-NMDAR encephalitis was smaller than that in HC group. Mousa Zidan et al. found that thalamic atrophy occurred in patients with AD and mild cognitive impairment (MCI), and that the decrease in thalamic gray matter volume was associated with cognitive decline in MCI patients at the early stage of the disease, which can be used as a biomarker to predict the progression from MCI to dementia (25). Ming-ge Li et al. found that total thalamic volume and the volume of seven thalamic subregions decreased in patients with Parkinson's disease, which was related to cognitive function (26). Thalamic atrophy is also found in patients with schizophrenia and bipolar disorder (27). At the same time, in a case report, magnetic resonance imaging of anti-NMDAR encephalitis patients showed bilateral thalamic hyperintensity with limited diffusion (28), which is consistent with the results of this study. There are extensive structural connections between the thalamus and prefrontal lobe, hippocampus, and other structures (29, 30). Therefore, we speculate that thalamic atrophy plays an important role in the process of cognitive impairment in patients with NMDAR encephalitis.

Our results show that gray matter volume in the mPFC_L, STG_L, and left rectus gyrus shrinks in patients with anti-NMDAR encephalitis. These brain regions are important nodes of the default mode network (DMN), and DMN damage in patients with anti-NMDAR encephalitis has been previously confirmed (31). The mPFC is involved in several execution processes, including response inhibition, inhibition control, and task switching (32–34), and it can perform self-control, avoid short-sighted behaviors, appropriately regulate emotions and reactions, make decisions, and consider how to solve problems and their consequences for long-term goals. The superior temporal gyrus is a brain region closely involved in cognitive functions such as hearing and semantic understanding (35). During a 9-month follow-up, frontal cortical atrophy was found in patients with anti-NMDAR encephalitis (15). In a study of quantitative and qualitative analysis of gray matter in patients with anti-NMDAR encephalitis, the temporal lobe was the primary region exhibiting atrophy, and 74% of the patients had epileptic activity (36). The common abnormalities in PET imaging include hypermetabolism in the frontal and temporal lobes, and studies have shown that the degree of frontotemporal lobe damage is positively correlated with the severity of the disease (10, 13, 37). As previously reported, atrophy is mainly seen in the frontotemporal region in anti-NMDAR encephalitis patients. This region includes a high density of NMDA receptors, and the binding epitope of the antibody is part of the NR1 subunit of NMDARs on the postsynaptic dendrites of the forebrain and hippocampus (38, 39). This may be the immunological cause of cognitive impairment in patients with anti NMDAR encephalitis.

Follow-up reports of anti-NMDAR encephalitis are somewhat controversial, as some studies observed reversible diffuse brain atrophy in patients, but others reported that atrophy in some regions was irreversible. Therefore, this study divided patients into NMDARE_SD and NMDARE_LD groups to study whether there are differences in the structure of brain gray matter in patients with different disease courses. We found that, compared with the NMDARE_SD group, gray matter volume of the posterior cerebellum and the precuneus was decreased in the NMDARE_LD group, and gray matter volume in the anterior and posterior cerebellar lobes was negatively correlated with the course of disease. Cerebellar projections pass through the thalamus and into the prefrontal lobe, sensorimotor area, visual and auditory cortex, etc., and participate in motor control and regulation of advanced cognitive functions. Cerebellar posterior lobe injury can lead to cerebellar cognitive-affective syndrome, which is characterized by deficits in executive function, visual spatial processing, language skills, and emotional regulation. The relationship between the cerebellum and cognitive function has been confirmed (40, 41). A follow-up study of anti-NMDAR encephalitis for more than 10 years found that some patients had reversible diffuse atrophy in some regions, but that cerebellar atrophy was irreversible (42). The precuneus is an important DMN node, which is closely related to self-cognition, monitoring the surrounding environment, introspection, and modulating the interaction between cognition and emotion (43). A previous study assessing cortical thickness reported DMN gray matter atrophy in patients with anti NMDAR encephalitis (44). The above results were not corrected for multiple comparisons, although a recent meta-analysis based of resting state fMRI data showed that strict multiple comparison correction could not increase the reliability of the results in a single study with small samples (45). Therefore, one should be cautious when interpreting the findings from subgroup analysis, and independent large samples and longitudinal self-control study are needed to confirm this observation in the future. The purpose of the cross-sectional study between NMDARE_LD and NMDARE_SD groups was to explore the gray matter atrophy pattern of patients with anti-NMDAR encephalitis at different diseases stages, which may serve as the basis for a longitudinal cohort study.

In the correlation analysis, we found a negative correlation between EC scores and gray matter volume of the mPFC_L and right thalamus, and a positive correlation between STG_L volume and cognitive ability. There are many fibrous connections between the frontal lobe, the temporal lobe, and the thalamus (29, 30, 46). When these brain regions atrophied significantly, the cognitive function, including memory and executive function, became worse. A study analyzed T1W MRI images of nearly 800 patients with anti-NMDAR encephalitis and found that abnormal sites were concentrated in the temporal lobe, frontal lobe, and thalamus, among others (47). This is consistent with the current findings, further supporting the accuracy of our results. The HAMD and HAMA scores of anti-NMDAR encephalitis decreased significantly, which indicated that the patients had anxiety and depression. Zhang et al. (48) showed that patients with anti-NMDAR encephalitis had depressive symptoms and the risk of suicide was higher than normal people, which suggesting that we should pay close attention to their emotional abnormalities in the process of treatment and rehabilitation. However, to our surprise, there is no significant correlation between VBM differential brain regions and HAMD and HAMA scores, which may be due to a variety of mental symptoms of anti-NMDAR encephalitis, or HAMD and HAMA cannot fully reflect the unique mental symptoms of patients.

Despite many meaningful findings in our study, there are still some limitations. (1) The course of disease of the patients is inconsistent, and different courses of disease may affect the results. Although we conducted further analysis in patients with different courses of disease to evaluate the progression of anti-NMDAR encephalitis, the results did not pass the multiple comparison correction test, even if the FWE/ False Discovery Rate (FDR)/Gaussian random fields (GRF)/Alphasim correction was calculated. These results can only be used as an exploratory analysis, and further longitudinal studies are still needed to corroborate the current findings. (2) Different etiologies, clinical manifestations, and treatment plans in patients may have a certain impact on the outcome, and their impact on our results cannot be ruled out at present. (3) The sample size is relatively small, and a larger sample size survey is needed to verify our results in the future. Therefore, future research should continue to expand the sample size and improve the experimental design to minimize these limitations.

## Conclusions

In conclusion, the current study confirmed that there was gray matter atrophy in anti-NMDAR encephalitis patients using VBM analysis. Gray matter atrophy was observed in the bilateral thalamus, frontal lobe, and temporal lobe. The gray matter atrophy of thalamus and frontal lobe is related to a decline in executive function, while that of temporal lobe is related to cognitive impairment. In addition, compared with the patients with short courses of disease, patients with long courses of disease demonstrated atrophy of the posterior cerebellar lobe and precuneus lobe. These findings highlight the necessity of observation and follow-up in convalescent patients with anti-NMDAR encephalitis to prevent persistent damage to their cognitive function. Overall, these results may help us better understand the potential pathophysiological mechanism of anti-NMDAR encephalitis.

## Data availability statement

The original contributions presented in the study are included in the article/supplementary material, further inquiries can be directed to the corresponding author/s.

## Ethics statement

The study protocol was approved by the First Affiliated Hospital of Guangxi Medical University Ethics Committee. The patients/participants provided their written informed consent to participate in this study.

## Author contributions

QL: writing–original draft and experimental design. JZha and CL: data curation. KS: investigation. ZL and BF: methodology. JZhe: funding acquisition. All authors finally agreed to publish this manuscript.

## Funding

This research was supported by the National Natural Science Foundation of China (contract authorization number: 81560223) and Guangxi Natural Science Foundation (contract authorization number: 2018GXNSFAA050149).

## Conflict of interest

The authors declare that the research was conducted in the absence of any commercial or financial relationships that could be construed as a potential conflict of interest.

## Publisher's note

All claims expressed in this article are solely those of the authors and do not necessarily represent those of their affiliated organizations, or those of the publisher, the editors and the reviewers. Any product that may be evaluated in this article, or claim that may be made by its manufacturer, is not guaranteed or endorsed by the publisher.

## References

[1] Dalmau J Graus F. Antibody-mediated encephalitis. N Engl J Med. (2018) 378:840–51. 10.1056/NEJMra1708712 29490181

[2] Deng S Qiu K Liu H Wu X Lei Q Lu W. Clinical characteristics and short-term prognosis of autoimmune encephalitis: a single-center cohort study in Changsha, China. Front Neurol. (2019) 10:539. 10.3389/fneur.2019.00539 31178819 PMC6543891

[3] Gable MS Sheriff H Dalmau J Tilley DH Glaser CA. The frequency of autoimmune N-methyl-D-aspartate receptor encephalitis surpasses that of individual viral etiologies in young individuals enrolled in the California Encephalitis Project. Clin Infect Dis. (2012) 54:899–904. 10.1093/cid/cir1038 22281844 PMC3297648

[4] Huang Q Xie Y Hu Z Tang X. Anti-N-methyl-D-aspartate receptor encephalitis: a review of pathogenic mechanisms, treatment, prognosis. Brain Res. (2020) 1727:146549. 10.1016/j.brainres.2019.146549 31726044

[5] Dalmau J Gleichman AJ Hughes EG Rossi JE Peng X Lai M. Anti-NMDA-receptor encephalitis: case series and analysis of the effects of antibodies. Lancet Neurol. (2008) 7:1091–8. 10.1016/s1474-4422(08)70224-2 18851928 PMC2607118

[6] Titulaer MJ McCracken L Gabilondo I Armangué T Glaser C Iizuka T. Treatment and prognostic factors for long-term outcome in patients with anti-NMDA receptor encephalitis: an observational cohort study. Lancet Neurol. (2013) 12:157–65. 10.1016/s1474-4422(12)70310-1 23290630 PMC3563251

[7] McKeon GL Robinson GA Ryan AE Blum S Gillis D Finke C. Cognitive outcomes following anti-N-methyl-D-aspartate receptor encephalitis: a systematic review. J Clin Exp Neuropsychol. (2018) 40:234–52. 10.1080/13803395.2017.1329408 28585453

[8] McKeon GL Scott JG Spooner DM Ryan AE Blum S Gillis D. Cognitive and social functioning deficits after anti-N-methyl-D-aspartate receptor encephalitis: an exploratory case series. J Int Neuropsychol Soc: JINS. (2016) 22:828–38. 10.1017/s1355617716000679 27546201

[9] Dalmau J Armangué T Planagumà J Radosevic M Mannara F Leypoldt F. An update on anti-NMDA receptor encephalitis for neurologists and psychiatrists: mechanisms and models. Lancet Neurol. (2019) 18:1045–57. 10.1016/s1474-4422(19)30244-3 31326280

[10] Bacchi S Franke K Wewegama D Needham E Patel S Menon D. Magnetic resonance imaging and positron emission tomography in anti-NMDA receptor encephalitis: a systematic review. J Clin Neurosci. (2018) 52:54–9. 10.1016/j.jocn.2018.03.026 29605275

[11] Zhang T Duan Y Ye J Xu W Shu N Wang C. Brain MRI characteristics of patients with anti-N-methyl-D-aspartate receptor encephalitis and their associations with 2-year clinical outcome. AJNR Am J Neuroradiol. (2018) 39:824–29. 10.3174/ajnr.A5593 29567651 PMC7410660

[12] Cai L Liang Y Huang H Zhou X Zheng J. Cerebral functional activity and connectivity changes in anti-N-methyl-D-aspartate receptor encephalitis: a resting-state fMRI study. NeuroImage Clin. (2020) 25:102189. 10.1016/j.nicl.2020.102189 32036276 PMC7013171

[13] Kerik-Rotenberg N Diaz-Meneses I Hernandez-Ramirez R Muñoz-Casillas R Reynoso-Mejia CA Flores-Rivera J. A metabolic brain pattern associated with anti-N-methyl-D-aspartate receptor encephalitis. Psychosomatics. (2020) 61:39–48. 10.1016/j.psym.2019.08.007 31611047

[14] Kataoka H Sawa N Tonomura Y Ueno S. Early progression of brain atrophy in patients with anti-N-methyl-D-aspartate receptor encephalitis: case reports. Medicine. (2017) 96:e6776. 10.1097/md.0000000000006776 28445312 PMC5413277

[15] Laurikainen H Isotupa I Nyman M Ilonen T Nummelin T Salokangas RKR. Longitudinal brain morphology in anti-NMDA receptor encephalitis: a case report with controls. BMC Psychiatry. (2019) 19:145. 10.1186/s12888-019-2141-4 31077184 PMC6511133

[16] Ashburner J Friston KJ. Voxel-based morphometry—the methods. Neuroimage. (2000) 11:805–21. 10.1006/nimg.2000.0582 10860804

[17] Hirakawa N Kuga H Hirano Y Sato J Oribe N Nakamura I. Neuroanatomical substrate of chronic psychosis in epilepsy: an MRI study. Brain Imaging Behav. (2020) 14:1382–87. 10.1007/s11682-019-00044-4 30734915 PMC7572341

[18] Donzuso G Monastero R Cicero CE Luca A Mostile G Giuliano L. Neuroanatomical changes in early Parkinson's disease with mild cognitive impairment: a VBM study; the Parkinson's Disease Cognitive Impairment Study (PaCoS). Neurol Sci. (2021). 10.1007/s10072-020-05034-9 33447925

[19] Matsuda HMRI. Morphometry in Alzheimer's disease. Ageing Res Rev. (2016) 30:17–24. 10.1016/j.arr.2016.01.003 26812213

[20] Kim GW Kim YH Jeong GW. Whole brain volume changes and its correlation with clinical symptom severity in patients with schizophrenia: a DARTEL-based VBM study. PLoS ONE. (2017) 12:e0177251. 10.1371/journal.pone.0177251 28520743 PMC5435302

[21] Wagner J Weber B Elger CE. Early and chronic gray matter volume changes in limbic encephalitis revealed by voxel-based morphometry. Epilepsia. (2015) 56:754–61. 10.1111/epi.12968 25809952

[22] Graus F Titulaer MJ Balu R Benseler S Bien CG Cellucci T. A clinical approach to diagnosis of autoimmune encephalitis. Lancet Neurol. (2016) 15:391–404. 10.1016/s1474-4422(15)00401-9 26906964 PMC5066574

[23] Fan J Gu X Guise KG Liu X Fossella J Wang H. Testing the behavioral interaction and integration of attentional networks. Brain Cogn. (2009) 70:209–20. 10.1016/j.bandc.2009.02.002 19269079 PMC2674119

[24] Halassa MM Kastner S. Thalamic functions in distributed cognitive control. Nat Neurosci. (2017) 20:1669–79. 10.1038/s41593-017-0020-1 29184210

[25] Zidan M Boban J Bjelan M Todorović A Stankov Vujanić T Semnic M. Thalamic volume loss as an early sign of amnestic mild cognitive impairment. J Clin Neurosci. (2019) 68:168–73. 10.1016/j.jocn.2019.07.004 31324472

[26] Li MG He JF Liu XY Wang ZF Lou X Ma L. Structural and functional thalamic changes in Parkinson's disease with mild cognitive impairment. J Magnet Reson Imaging: JMRI. (2020) 52:1207–15. 10.1002/jmri.27195 32557988

[27] Lee DK Lee H Park K Joh E Kim CE Ryu S. Common gray and white matter abnormalities in schizophrenia and bipolar disorder. PLoS ONE. (2020) 15:e0232826. 10.1371/journal.pone.0232826 32379845 PMC7205291

[28] Dubey S Ghosh R Dubey MJ Sengupta S Benito-León J Ray BK. Bilateral thalamic changes in anti-NMDAR encephalitis presenting with hemichorea and dystonia and acute transient psychotic disorder. J Neuroimmunol. (2020) 347:577329. 10.1016/j.jneuroim.2020.577329 32745805 PMC7374132

[29] Parnaudeau S Bolkan SS Kellendonk C. The mediodorsal thalamus: an essential partner of the prefrontal cortex for cognition. Biol Psychiatry. (2018) 83:648–56. 10.1016/j.biopsych.2017.11.008 29275841 PMC5862748

[30] Kafkas A., Mayes, A. R., and Montaldi, D. Thalamic-medial temporal lobe connectivity underpins familiarity memory. Cereb Cortex. (2020) 30:3827–37. 10.1093/cercor/bhz345 31989161 PMC7232995

[31] Peer M Prüss H Ben-Dayan I Paul F Arzy S Finke C. Functional connectivity of large-scale brain networks in patients with anti-NMDA receptor encephalitis: an observational study. Lancet Psychiatry. (2017) 4:768–74. 10.1016/s2215-0366(17)30330-9 28882707

[32] Yuan P Raz N. Prefrontal cortex and executive functions in healthy adults: a meta-analysis of structural neuroimaging studies. Neurosci Biobehav Rev. (2014) 42:180–92. 10.1016/j.neubiorev.2014.02.005 24568942 PMC4011981

[33] Chen CY Muggleton NG Tzeng OJ Hung DL Juan CH. Control of prepotent responses by the superior medial frontal cortex. NeuroImage. (2009) 44:537–45. 10.1016/j.neuroimage.2008.09.005 18852054

[34] Rushworth MF Hadland KA Paus T Sipila PK. Role of the human medial frontal cortex in task switching: a combined fMRI and TMS study. J Neurophysiol. (2002) 87:2577–92. 10.1152/jn.2002.87.5.2577 11976394

[35] Mellem MS Jasmin KM Peng C Martin A. Sentence processing in anterior superior temporal cortex shows a social-emotional bias. Neuropsychologia. (2016) 89:217–24. 10.1016/j.neuropsychologia.2016.06.019 27329686 PMC5384858

[36] Bassal FC Harwood M Oh A Lundberg JN Hoffman J Cornejo P. Anti-NMDA receptor encephalitis and brain atrophy in children and adults: a quantitative study. Clin Imaging. (2021) 78:296–300. 10.1016/j.clinimag.2021.05.028 34186471

[37] Leypoldt F Buchert R Kleiter I Marienhagen J Gelderblom M Magnus T. Fluorodeoxyglucose positron emission tomography in anti-N-methyl-D-aspartate receptor encephalitis: distinct pattern of disease. J Neurol Neurosurg Psychiatry. (2012) 83:681–6. 10.1136/jnnp-2011-301969 22566598 PMC3740122

[38] Dalmau J Tüzün E Wu HY Masjuan J Rossi JE Voloschin A. Paraneoplastic anti-N-methyl-D-aspartate receptor encephalitis associated with ovarian teratoma. Ann Neurol. (2007) 61:25–36. 10.1002/ana.21050 17262855 PMC2430743

[39] Dalmau J Lancaster E Martinez-Hernandez E Rosenfeld MR Balice-Gordon R. Clinical experience and laboratory investigations in patients with anti-NMDAR encephalitis. Lancet Neurol. (2011) 10:63–74. 10.1016/s1474-4422(10)70253-2 21163445 PMC3158385

[40] Molinari M Filippini V Leggio MG. Neuronal plasticity of interrelated cerebellar and cortical networks. Neuroscience. (2002) 111:863–70. 10.1016/s0306-4522(02)00024-6 12031409

[41] Tedesco AM Chiricozzi FR Clausi S Lupo M Molinari M Leggio MG. The cerebellar cognitive profile. Brain. (2011) 134 (Pt 12):3672–86. 10.1093/brain/awr266 22036960

[42] Iizuka T Kaneko J Tominaga N Someko H Nakamura M Ishima D. Association of progressive cerebellar atrophy with long-term outcome in patients with anti-N-methyl-d-aspartate receptor encephalitis. JAMA Neurol. (2016) 73:706–13. 10.1001/jamaneurol.2016.0232 27111481 PMC5018902

[43] Deng D Liao H Duan G Liu Y He Q Liu H. Modulation of the default mode network in first-episode, drug-Naïve major depressive disorder via acupuncture at Baihui (GV20) acupoint. Front Hum Neurosci. (2016) 10:230. 10.3389/fnhum.2016.00230 27242492 PMC4869560

[44] Xu J Guo Y Li J Lv X Zhang J Zhang J. Progressive cortical and sub-cortical alterations in patients with anti-N-methyl-D-aspartate receptor encephalitis. J Neurol. (2021). 10.1007/s00415-021-10643-1 34297178

[45] Jia X-Z Zhao N Dong H-M Sun J-W Barton M Burciu R. Small P values may not yield robust findings: an example using REST-meta-PD. Sci Bull. (2021) 66:2148–52. 10.1016/j.scib.2021.06.007 36654102

[46] Leszczyński M Staudigl T. Memory-guided attention in the anterior thalamus. Neurosci Biobehav Rev. (2016) 66:163–5. 10.1016/j.neubiorev.2016.04.015 27130694

[47] Heine J Prüss H Bartsch T Ploner CJ Paul F Finke C. Imaging of autoimmune encephalitis—relevance for clinical practice and hippocampal function. Neuroscience. (2015) 309:68–83. 10.1016/j.neuroscience.2015.05.037 26012492

[48] Zhang L Sander JW Zhang L Jiang XY Wang W Shuang K. Suicidality is a common and serious feature of anti-N-methyl-D-aspartate receptor encephalitis. J Neurol. (2017) 264:2378–86. 10.1007/s00415-017-8626-5 28993875

